# Postoperative pain after single and multiple visits for root canal therapy in necrotic teeth

**DOI:** 10.6026/973206300212157

**Published:** 2025-07-31

**Authors:** Dinesh Govinda Kamath, Blesy Koshy Varughese, Treesa William Gomez, Sneha Lembai, Mary V. Gibi, Justin Eldho

**Affiliations:** 1 Department of Conservative Dentistry & Endodontics, Indira Gandhi Institute of Dental Sciences, Nellikuzhi, Kothamangalam, Ernakulam, Kerala, India; 2 Department of Prosthodontics and Crown and Bridge Indira Gandhi Institute of Dental Sciences, Nellikuzhi, Kothamangalam, Ernakulam, Kerala, India; 3 Consultant & Private Practitioner in Conservative Dentistry and Endodontics, Kamath's Dental Clinic, Ernakulam, Kerala, India

**Keywords:** Postoperative pain, single-visit endodontics, multiple-visit endodontics, necrotic teeth, root canal therapy, visual analog scale (vas), calcium hydroxide, endodontic treatment, pain assessment, randomized clinical trial

## Abstract

Postoperative pain remains a key concern in root canal therapy for necrotic teeth, with ongoing debate over the optimal number of treatment visits. This randomized clinical trial compared pain outcomes between single-visit and multiple-visit endodontic treatments in 80 patients. Pain was assessed using the Visual Analog Scale at 6, 24, 48, and 72 hours postoperatively. Both groups showed a progressive decline in pain with no statistically significant difference at any time point. The results suggest that single-visit therapy is as effective as multiple-visit protocols in managing postoperative pain in necrotic teeth.

## Background:

Pain after surgery is typical and expected following root canal therapy in cases of dead pulps and disease in the surrounding bone tissue. It is important because it greatly affects how happy patients are and their feeling about the success of treatment. Postoperative pain is linked to different things such as bacterial contamination, methods used during surgery, cleaning techniques and the number of appointments needed [1, 2]. A common talked-about topic in endodontics is whether root canals should be done in one appointment or several, especially when the pulp is necrotic [3]. This technique is becoming popular because it saves time at the dentist, reduces chances of contamination and patients usually follow the treatment plan better [4]. Also, there exists some apprehension about the amount of bacteria being reduced and overly severe pain that might occur post-surgery in cases with severe infection [5]. Unlike single-visit endodontics, using several treatments and calcium hydroxide solutions is thought to keep infections in control and cut down on repeat symptoms [6, 7]. Because of its antimicrobial and dissolving effects on tissue, calcium hydroxide is a key part of multiple-visit therapy, although its impact on postoperative pain is less clear. To be fully effective, calcium hydroxide treatment takes seven days, but compliance and time for applying the material may affect the outcome [8]. Furthermore, if lots of instruments and irrigants are used in necrotic cases, it may lead to inflammation around the root, causing pain perception to increase, no matter the number of visits [9]. Despite new meta-analyses and systematic reviews on this, disparities in treatment plans, surgeon abilities, Evaluating pain and whether severe pain is defined in the same way result in mixed outcomes. Some research indicates that single-visit procedures and multiple-visit approaches do not vary much in terms of postoperative pain, yet others have pointed out that the multiple-visit protocol may lower pain at the early stages after surgery [10, 11]. Therefore, it is of interest to evaluate postoperative pain after single and multiple for visit root canal therapy in necrotic teeth.

## Materials and Methods:

This randomized clinical trial was conducted on 80 patients aged between 18 and 55 years who presented with single-rooted teeth diagnosed with pulpal necrosis.

## Patient selection and group allocation:

Participants were selected based on clinical and radiographic diagnosis confirming pulpal necrosis and the presence of a single root canal. Exclusion criteria included systemic illness, acute apical abscess, retreatment cases, pregnancy, and allergy to local anesthetics or prescribed analgesics. Using a computer-generated randomization list, patients were equally divided into two groups:

[1] Group A (n=40): Underwent root canal treatment in a single visit.

[2] Group B (n=40): Received root canal therapy in two visits with an interappointment dressing of calcium hydroxide.

## Clinical procedure:

All surgeries were carried out by a top endodontist with the area isolated by a rubber dam. Two percent lidocaine with 1:80,000 epinephrines was used to give local anesthesia. An apex locator was used to determine the length after access was obtained and this was finally checked on a radiograph. A rotary NiTi system was used to shape the root canal using the crown-down method. After planning and dissecting, the samples were irrigated with 3% sodium hypochlorite and saline and then a final clean was done with 17% EDTA. The dentist completed obturation for Group A during one visit by placing lateral gutta-percha and a resin-based sealer and restoring the tooth temporarily with glass ionomer cement. At the first visit for Group B, calcium hydroxide was used as a medicament and the canal was temporarily filled with composite before the next appointment. After the first week, the canal was cleaned, moisture applied and obturated as before for Group A.

## Post-surgery pain assessment:

The scale patients used was the Visual Analog Scale (VAS), in which 0 stands for no pain and 10 represents the most intense pain imaginable. Appraisals were done at 6, 24, 48 and 72 hours after the patient received treatment. Patients were told the same way to measure pain and were spoken to over the phone so that their pain scores could be recorded.

## Statistical analysis:

Using the SPSS software version 26, the data were organized and examined. Group differences for categorical variables were established using the Chi-square test and the scores from VAS in both groups were examined using repeated-measures ANOVA. A p - value lower than 0.05 was recognized as statistically significant.

## Results:

A total of 80 patients participated in the study, with 40 patients assigned to the single-visit group (Group A) and 40 to the multiple-visit group (Group B). The demographic distribution of participants is presented in Table 1 (see PDF). There were no statistically significant differences between the two groups with regard to age or gender (p > 0.05). Postoperative pain levels, measured using the Visual Analog Scale (VAS), were recorded at 6, 24, 48, and 72 hours. At the 6-hour mark, moderate to severe pain (VAS ≥4) was reported in 12 patients (30%) in Group A and in 9 patients (22.5%) in Group B (p = 0.38). At 24 hours, the pain intensity had reduced, with only 7 patients (17.5%) in Group A and 4 patients (10%) in Group B reporting VAS scores ≥4 (Table 2 - see PDF). The difference in pain intensity between the groups at each time point was not statistically significant (p > 0.05). A trend of progressive pain reduction was observed in both groups. By 48 hours, only 3 patients (7.5%) in Group A and 2 patients (5%) in Group B reported mild discomfort (VAS 1-3). At 72 hours, 1 patient (2.5%) in Group A continued to report mild pain, whereas none of the patients in Group B experienced any pain (Table 3 - see PDF). Repeated-measures ANOVA revealed a significant decrease in VAS scores over time within each group (p < 0.001); however, intergroup comparisons showed no statistically significant difference at any interval (p > 0.05). The time-wise distribution of mean VAS scores is illustrated in Table 4 (see PDF), confirming a similar pattern of pain resolution in both treatment modalities.

## Discussion:

Its purpose was to see how much and how often patients reported postoperative pain after single-day root canal treatment, compared to the same procedure spread out over several days. Pain levels kept decreasing in both groups after the operation, but there was no evident difference between the two groups at any point. This is in line with studies that have pointed out that the number of visits might not be the main factor affecting postoperative pain in cases of necrotic pulps [1, 2]. Several things contribute to endodontic postoperative pain like microbial presence, removal of debris from the root tip and inflammation around the tooth's root [3, 4]. Because diverse bacteria are often found in necrotic teeth, getting them all treated and cleaned is not easy, regardless of the number of appointments. Calcium hydroxide helps stop bacterial growth in the mouth, but it is not yet certain if it helps with postoperative pain when used in multiple visits [5, 6, 7-8]. One possible reason for no difference in pain scores is that all cases used rotary instruments and proper irrigation. The use of modern endodontic systems along with sodium hypochlorite and EDTA makes it possible to reduce most microbes in just one visit which often means no need for medicaments between appointments [9, 10]. The healing of an apex and the reduction of pain are greatly affected by the obturation technique, the durability of the seal and the type of restoration placed at the chewing surface [11]. Postoperative pain outcomes are generally comparable between single-visit and multiple-visit root canal treatments [12]. Some evidence indicates that single-visit treatment may reduce short-term postoperative discomfort. Variability in study designs and pain assessment methods contributes to inconsistent findings across research [13].

Also, feeling anxious, past experiences of pain or being from a particular gender could change how pain is perceived and such factors were not part of this study. While researchers wanted the treatments and operators to be standardized, variability in the healing responses of patients always remains a factor in clinical trials [14]. Not considering multirooted teeth is another limitation since these teeth may heal differently because of their complex structure. Clinically, it means endodontic treatments in one visit are reliable and worth considering whenever scheduling, compliance or time issues are present in necrotic cases. But in cases with continuing infections, evident inflammation or tooth anatomy that makes disinfection slow, adopting a protocol with multiple visits can be useful [15]. Apart from microbes, how the infection is surgically accessed and irrigated is very important for the healing of the area and for reducing postoperative pain. All cavity preparation steps and irrigation used rotary NiTi files combined with hypochlorite which has been proven to reduce the getting of dental material to the apex and successfully clean the cavity [6]. From studies, we know that rotary instrumentation systems, because they control how cuts are made and waste is handled, may lead to less pain after surgery than the manual method [7]. The final step of using EDTA in irrigation could have helped remove the smear layer, let the sealer penetrate better and decrease the number of bacteria left on the surface [8]. It is very important to ensure both effective obturation and secure sealing of coronal margins, because inadequate sealing can allow bacteria to enter the tooth and cause ongoing problems and pain. The reason gutta-percha with resin-based sealers is still preferred is that they fit the criteria of being safe and effective at sealing [9]. In addition, quick placement of a temporary or permanent crown greatly reduces the possible microleakage problem which is linked to extra pain and unsuccessful endodontic treatment after surgery [2]. Similar filling and restoration were given to participants in both groups which are expected to reduce any problems caused by coronal leakage. It is also necessary to look at the mental and behavioral aspects of pain. How someone experiences pain when getting dental treatment depends on their own sensitivity, feelings and prior dental history [3]. Even though PROMs like anxiety and fear were not measured here, they might be the reason for some of the variation in participants' VAS scores. Studies in the future should recognize these factors to gain a fuller picture of pain during endodontic treatment. Even with its gaps, this study proves that using proper techniques, a single trip to the dentist for treating dead teeth is as good and safe as going for multiple visits.

## Conclusion:

Single-visit and multiple-visit root canal therapies result in comparable levels of postoperative pain in necrotic teeth. The choice of treatment should be based on clinical judgment, case complexity, and patient preference. Single-visit endodontics is a reliable and efficient option when conditions permit.
